# The outside medical evacuation for cancer from Madagascar

**DOI:** 10.11604/pamj.2019.34.73.19374

**Published:** 2019-10-04

**Authors:** Refeno Valéry, Hasiniatsy Nomeharisoa Rodrigue Emile, Ramahandrisoa Andriatsihoarana Voahary Nasandratriniavo, Rabarijaona Léontine Irène, Rafaramino Florine

**Affiliations:** 1 Oncology Department of Professor Zafisaona Gabriel Teaching Hospital, Faculty of Medicine of Mahajanga, Mahajanga, Madagascar; 2 Oncology and Palliative Care Department of Military Hospital, Faculty of Medicine of Antananarivo, Antananarivo, Madagascar; 3 Oncology Department of Joseph Ravoahangy Andrianavalona Teaching Hospital, Faculty of Medicine of Antananarivo, Antananarivo, Madagascar

**Keywords:** Cancer, medical evacuation, Madagascar, technical platforms

## To the editors of the Pan African Medical Journal

Medical evacuation (MEDEVAC) is the transfer of a patient from one health facility to another. This patient is suffering from a medical or surgical condition requiring investigation and/or care exceeding the limits of the capacity and technical competence of the health unit that transfers [1]. In developed countries, cancer care benefits from the availability and accessibility of different therapeutic resources [2]. In developing countries, particularly those in Africa, the management of cancer is hampered by the unavailability and inaccessibility of certain technical platforms including radiotherapy and scintigraphy [3]. Knowledge of the missing technical platforms could help prioritize investments in health infrastructure for the fight against cancer. Thus, our objective was to describe the requests for external medical evacuation for cancer in order to make an inventory of the technical platforms missing in Madagascar in terms of oncology.

We carried out a retrospective cross-sectional descriptive study, at the Department of the Hospital System of the Ministry of Public Health, Antananarivo, which is the only institution authorized to grant the outside medical evacuation in Madagascar, from 1^st^ January 2012 to 31^st^ December 2012. We have included all files of external medical evacuation request. After excluding records of non-oncology cases, we retained patient records requesting outside medical evacuation for cancer. Of the 91 external MEDEVAC application files, 25 were cancer cases (27.47%). The mean age of the patients was 45.12±18.11 years and the sex ratio was 0.47. Eighteen patients (72%) came from the capital of Madagascar. Metropolitan France was the most popular MEDEVAC site (44%). The most represented clinical situations were breast cancer waiting for adjuvant treatment (n=9 or 36%), lymphoma waiting for second-line treatment (n=2 or 8%) and brain tumors waiting for initial treatment (n=2 or 8%). The main technical platforms required were radiotherapy (n=13 or 52%), specialized paraclinical investigations (n=5 or 20%), specialized surgeries (n=4 or 16%) and specialized medical treatments (n=3 or 12%). Table 1 summarizes in detail the reasons for requesting outside medical evacuation for cancer. The most common clinical situations were breast cancer waiting for adjuvant treatment (36%), lymphoma waiting for second line treatment (8%) and brain tumors waiting for initial treatment (8%). Only one patient had requested outside MEDEVAC for adjuvant treatment of cervical cancer. This fact draws our attention because cervical cancer was, with breast cancer, among the most represented cancers in epidemiological studies conducted in the cancer centers of the capital [4-7]. According to GLOBOCAN 2012 estimates, cervical cancer is the most common cancer in Madagascar [8]. Otherwise, radiotherapy was an essential treatment in the management of cervical cancer in Madagascar when it was functional [2]. Therefore, in the absence of radiotherapy, it can be assumed that the majority of patients with cervical cancer did not receive optimal treatment.

**Table 1:** Summary of the reasons for requesting outside medical evacuation for cancer in Madagascar in 2012

Patient	Clinical situation	Place of evacuation	Technical platform requested
01	Remission assessment of kidney cancer	France	Positon Emission Tomography (PET)
02	Initial treatment of esophageal cancer	France	Specialized surgery of the esophagus
03	Pulmonary tumor not accessible to bronchial fibroscopy	France	Biopsy guided by computed tomography
04	Adjuvant treatment of Kaposi's sarcoma	France	Radiotherapy
05	Adjuvant treatment of cancer of the base of the tongue	Reunion	Radiotherapy
06	Adjuvant treatment for breast cancer	France	Radiotherapy
07	Treatment of Multiple Myeloma Relapse	France	Hematopoietic stem cell transplantation
08	Complementary treatment of lung cancer	Mauritius	Radiotherapy
09	Adjuvant treatment for breast cancer	Reunion	Radiotherapy
10	Adjuvant treatment for breast cancer	Mauritius	Radiotherapy
11	Surgical treatment of a cholangiocarcinoma	Reunion	Specialized liver surgery
12	Adjuvant treatment for breast Cancer	Reunion	Radiotherapy
13	Adjuvant treatment for breast Cancer	Reunion	Radiotherapy
14	Adjuvant treatment for breast Cancer	Reunion	Radiotherapy
15	Complementary treatment of cervical cancer	Reunion	Radiotherapy
16	Esophageal tumor investigation	France	Esophageal echo-endoscopy
17	Remission assessment of a mediastinal tumor	France	Positon Emission Tomography
18	Adjuvant treatment for breast Cancer	Reunion	Radiotherapy
19	Initial treatment of brain cancer	Mauritius	Specialized surgery of brain
20	Adjuvant treatment for breast Cancer	Reunion	Radiotherapy
21	Treatment of lymphoma relapse	France	Intensive chemotherapy + Hematopoietic stem cell transplantation
22	Treatment of lymphoma relapse	Reunion	Intensive chemotherapy + Hematopoietic stem cell transplantation
23	Adjuvant Treatment for breast Cancer	Reunion	Radiotherapy
24	Treatment of a recurrence of skin cancer	France	Specialized surgery of the hand
25	Initial treatment of brain cancer	France	Specialized brain surgery

In our study, the main technical platform requested was radiotherapy (52%). Outside MEDEVAC patterns seem to vary according to the technical platform available within the country. In the study by Amoussou-Guenou *et al.* in Benin, the main reasons for outside evacuation from 2006 to 2010 were radiotherapy and scintigraphy [3]. The scintigraphy was functional in Madagascar in 2012 and did not require outside MEDEVAC. Currently, this treatment is available based on the supply of radioactive material. In the Muteganya *et al.* study in Burundi from 1986 to 1993, radiotherapy-being unavailable-was the only reason for requesting outside MEDEVAC for cervical cancer patients who needed it [9]. According to Barbe *et al.* the specialties of onco-hematology, neurosurgery, cardiac surgery and radiotherapy did not exist in New Caledonia from 2008 to 2011 and required the outside medical evacuation of patients concerned by these diseases. In addition, New Caledonia received outside MEDEVAC from neighboring islands, particularly from the Wallis and Futuna Islands, for the management of certain serious medical and surgical conditions and all cancers since chemotherapy was not available in these areas [10]. Many chemotherapy molecules and neurosurgery were available in Madagascar and have not motivated outside MEDEVAC in our sample. The overwhelming demand of MEDEVAC for radiotherapy of our patients can be explained by the fact that radiotherapy was not available in Madagascar from 2009 to 2014 [2]. The existence of a private radiotherapy center that has been operational since April 2015 should reduce the number of outside medical evacuations for cancer and improve the management of patients who need it. In addition, a second radiotherapy center is currently under construction in a Teaching Hospital of the capital and should further improve access to this treatment. Nevertheless, a lot of authorities investment remains to be done because positron emission tomography, digestive echo-endoscopy, interventional radiology and hematopoietic stem cell transplantation which were unavailable in 2012 are still unavailable in 2018 and require the outside MEDEVAC of patients.

## Competing interests

The authors declare no competing interests.
